# *i*PhyDSDB: Phytoplasma Disease and Symptom Database

**DOI:** 10.3390/biology13090657

**Published:** 2024-08-24

**Authors:** Wei Wei, Jonathan Shao, Yan Zhao, Junichi Inaba, Algirdas Ivanauskas, Kristi D. Bottner-Parker, Stefano Costanzo, Bo Min Kim, Kailin Flowers, Jazmin Escobar

**Affiliations:** 1 Molecular Plant Pathology Laboratory, Beltsville Agricultural Research Center, Agricultural Research Service, United States Department of Agriculture, Beltsville, MD 20705, USA; yan.zhao@usda.gov (Y.Z.); junichi.inaba@usda.gov (J.I.); algirdas.ivanauskas@usda.gov (A.I.); kristi.bottner@usda.gov (K.D.B.-P.); stefano.costanzo@usda.gov (S.C.); bomin.kim@usda.gov (B.M.K.); kailin.flowers@usda.gov (K.F.); jazmin.escobar@usda.gov (J.E.); 2 Statistics and Bioinformatics Group—Northeast Area, Agricultural Research Service, United States Department of Agriculture, Beltsville, MD 20705, USA; jonathan.shao@usda.gov

**Keywords:** NCBI nucleotide database, symptom recognition, advanced search functionality, disease diagnosis, image-based AI detection

## Abstract

**Simple Summary:**

Phytoplasmas are minute bacteria that infect many plant species, causing economic losses and impacting agriculture. Early diagnosis is crucial to effective disease management. A symptom database, *i*PhyDSDB, was developed by retrieving nearly 35,000 phytoplasma DNA sequences from the NCBI nucleotide database and identifying 945 plant species associated with phytoplasma diseases. The database includes curated links to symptom images and detailed disease information. Implemented on a web-based interface using MySQL and PHP, *i*PhyDSDB features advanced search functionality. This tool helps farmers, growers, researchers, and educators efficiently query based on plant host and symptom type, aiding in the identification and management of phytoplasma-related diseases.

**Abstract:**

Phytoplasmas are small, intracellular bacteria that infect a vast range of plant species, causing significant economic losses and impacting agriculture and farmers’ livelihoods. Early and rapid diagnosis of phytoplasma infections is crucial for preventing the spread of these diseases, particularly through early symptom recognition in the field by farmers and growers. A symptom database for phytoplasma infections can assist in recognizing the symptoms and enhance early detection and management. In this study, nearly 35,000 phytoplasma sequence entries were retrieved from the NCBI nucleotide database using the keyword “phytoplasma” and information on phytoplasma disease-associated plant hosts and symptoms was gathered. A total of 945 plant species were identified to be associated with phytoplasma infections. Subsequently, links to symptomatic images of these known susceptible plant species were manually curated, and the Phytoplasma Disease Symptom Database (*i*PhyDSDB) was established and implemented on a web-based interface using the MySQL Server and PHP programming language. One of the key features of *i*PhyDSDB is the curated collection of links to symptomatic images representing various phytoplasma-infected plant species, allowing users to easily access the original source of the collected images and detailed disease information. Furthermore, images and descriptive definitions of typical symptoms induced by phytoplasmas were included in *i*PhyDSDB. The newly developed database and web interface, equipped with advanced search functionality, will help farmers, growers, researchers, and educators to efficiently query the database based on specific categories such as plant host and symptom type. This resource will aid the users in comparing, identifying, and diagnosing phytoplasma-related diseases, enhancing the understanding and management of these infections.

## 1. Introduction

Phytoplasmas are small, phloem-restricted bacteria that infect various plant species [1,2,3]. Phytoplasmas are transmitted from one plant to another by insect vectors, mainly leafhoppers, planthoppers, and psyllids [4]. In many plant species, phytoplasma infection impacts both reproductive and vegetative growth, inducing floral and foliar symptoms [5,6,7,8,9]. Virescence, phyllody, and cauliflower-like inflorescence (CLI) are typical floral symptoms affecting reproductive growth, known as floral reversion [8,9,10]. Witches’- broom is a common foliar symptom characterized by excessive vegetative growth [11]. In addition, phytoplasma infections may not cause typical symptoms in some plant species, such as certain weeds, grass, and woody species [12,13]. However, these species serve as important reservoirs for phytoplasma transmission.

Phytoplasma diseases can significantly reduce crop quality and yield, leading to economic losses for farmers and reduced food security in affected regions [2]. Early and rapid diagnosis of phytoplasma infections is crucial for preventing the spread of these diseases and minimizing their impact on agriculture and the environment. Currently, diagnosing phytoplasma diseases relies heavily on laboratory molecular techniques and expert knowledge [3], making it challenging for farmers and growers to identify and diagnose phytoplasma diseases in a timely and accurate manner.

Disease and symptom databases have demonstrated effectiveness in assisting the diagnosis of various human diseases, as evidenced by studies like those by Wu et al. [14] and Sharma et al. [15]. Similarly, in plant pathology, platforms such as PlantVillage [16] (https://plantvillage.psu.edu/, accessed on 10 September 2023) and PlantDiseases.org (UC Berkeley, https://www.plantdiseases.org/, accessed on 10 September 2023) have also been developed. However, these existing plant disease databases lack comprehensive data on diseases associated with phytoplasma infections, especially phytoplasma-specific symptoms. In this study, we established a database that consists of various phytoplasma diseases and their associated symptoms, and we implemented it on a web-based interface (https://plantpathology.ba.ars.usda.gov/iphydsdb/iphydsdb.html, accessed on 23 May 2024). The database is called the Phytoplasma Disease and Symptom Database (*i*PhyDSDB), which includes 1264 links to symptomatic images collected from 372 out of 945 plant species that are associated with phytoplasma infections. This database can provide a reference point for matching symptoms to phytoplasma diseases, aiding in phytoplasma disease diagnosis. With access to a database of symptoms, growers and farmers can quickly recognize signs of disease and take proactive measures to prevent its spread before it causes significant damage. In addition, the ready accessibility of the database to farmers, growers, and researchers worldwide will greatly facilitate information sharing and the development of strategies to combat emerging and re-emerging phytoplasma diseases globally.

## 2. Materials and Methods

### 2.1. Collection of Information on Plant Hosts of Phytoplasmas and Their Associated Symptoms

**Figure 1:**
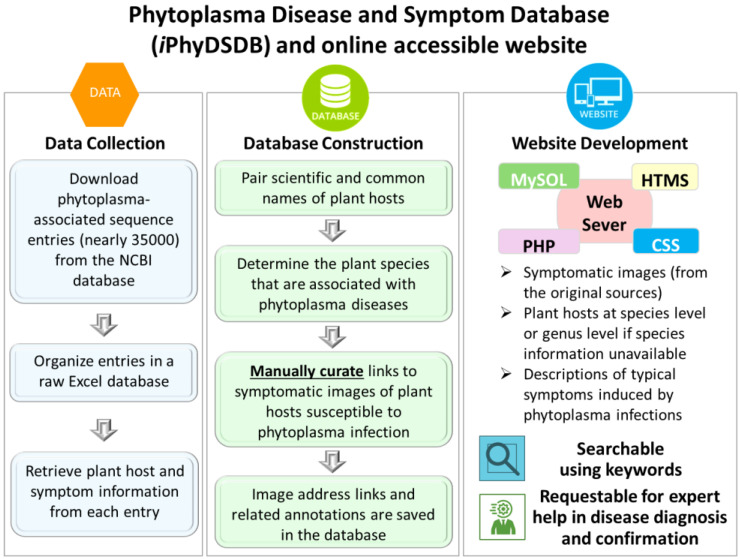
Diagram presenting the architecture, key features, and workflow involved in the construction of the Phytoplasma Disease and Symptom Database (*i*PhyDSDB) and website development.

Phytoplasma nucleotide sequence entries were retrieved from the NCBI nucleotide database using the keyword “phytoplasma,” and a raw database was established in Microsoft Excel (Microsoft Corporation, Seattle, WA, USA) to organize the sequence entries (Figure 1). Then, information on plant hosts was manually collected from the descriptions of each GenBank entry. Since sequence submitters often provided either common or scientific names inconsistently, several resources and tools were utilized to match plant common names with their corresponding scientific names, and vice versa. These resources and tools include Plants of the World Online (POWO, (https://powo.science.kew.org/, accessed on 20 December 2023)), The Plant List ((http://www.theplantlist.org/, accessed on 20 December 2023)), and the USDA PLANTS Database ((https://plants.usda.gov/home, accessed on 20 December 2023)). The paired common and scientific names were used to create a complete dataset. In addition, phytoplasma-induced symptom information was also manually extracted from the individual sequence entries and integrated into our dataset. Furthermore, to determine the plant host range of phytoplasma, plant host names were sorted out, and the duplicates were removed using the Excel filter function to finalize the list (the numbers and the names) of plant species known to be susceptible to phytoplasma infections.

### 2.2. Phytoplasma Disease and Symptom Database Construction and Website Development

Images of phytoplasma-infected plants were identified online, and the Internet addresses (URLs) of individual original images were embedded in the Phytoplasma Disease and Symptom Database (*i*PhyDSDB). An interactive web interface was constructed to query and display the data stored in the *i*PhyDSDB using MySQL Server 8.0.32 (MySQL AB, Uppsala, Sweden) as the back end. The front end was built using PHP 8.0.30, HTML, and CSS (Figure 1).

## 3. Results

### 3.1. Plant Host Range of Phytoplasmas

Phytoplasmas can infect a wide range of plant species; however, the exact number of plant species susceptible to phytoplasma infections is unknown. Some reports described that phytoplasmas could infect at least 700 plant species [17,18], while others indicated that the number may exceed 1000 plant species [19,20]. In this study, nearly 35,000 phytoplasma sequence entries were retrieved from the NCBI nucleotide database, and plant host and symptom information was collected from individual entries (Figure 1). A total of 945 known plant species and 68 unknown species (at the genus level) were found to be associated with phytoplasma infections.

### 3.2. Phytoplasma-Induced Symptoms

Numerous phytoplasma infection symptoms were described by the submitters of the GenBank entries that registered approximately 35,000 phytoplasma DNA sequences. The terms of the phytoplasma symptoms were curated from individual sequence entries and summarized as follows in alphabetical order:

Apical curl necrosis, big bud, blotchy-mottle, blue dwarf, blue yellows, bogia syndrome, branch-inducing, browning, bud proliferation, bunchy leaf, bunchy shoot, bunchy top, bushy stunt, chloranthy, chlorosis, chlorotic leafroll, cauliflower-like inflorescence (CLI), cluster, decline, deformation, dieback, disrupted sympodial growth pattern (DSGP), dwarf, false blossom, fasciation, flavescence dorée, flower abnormality, flower distortion, flower stunt, flower-inducing, frogskin, fruit proliferation, giant calyx, grassy shoot, green ear, green grassy shoot, green petal, leaf deformation, leaf rolling, leaf rot, leaflet flaccidity (root wilt), leafy fruit, lethal decline, lethal wilt, lethal yellowing, lethal yellows, little leaf, multiple inflorescence, multiplier, orange leaf, phyllody, purple top, purple top wilt, purple vein, red leaf, reddening, redness, reversion, root wilt, scorch, shoot proliferation, slow decline, small flowers, spear rot, stem curling and phyllody, stunting, sudden decline, virescence, white leaf, white tip dieback, whiteness, whitening, wilt, witches’-broom, wrinkled leaves and phyllody, yellow decline, yellow edge, yellow leaf, yellow leaf roll, yellow patch, yellowing, and yellows.

Although nearly 80 terms were found to specify symptoms, these can be grouped into several categories, including flower deformation, inflorescence abnormality, leaf color change, stunting, branching, and decline. For example, floral deformations include virescence, phyllody, big bud, giant calyx, small flowers, etc.

### 3.3. Typical Symptoms Induced by Phytoplasma Infections

**Figure 2:**
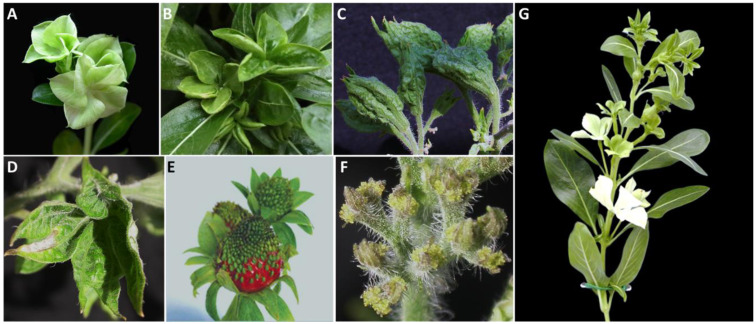
Phytoplasma infection-induced floral reversions that affect reproductive growth in plants. (**A**,**B**), virescence (**A**) and phyllody (**B**) in periwinkles. (**C**,**D**), big bud (**C**) and phyllody (**D**) symptoms in tomato plants. (**E**), phyllody symptoms in strawberry plants. This particular phyllody occurred in the infected carpel, also called carpel phylloid. (**F**), cauliflower-like inflorescence (CLI) in phytoplasma-infected tomato plants. Such inflorescence fails to produce normal flowers and set fruits. (**G**), multiple symptoms (virescence, phyllody, and big bud) occurred in the same periwinkle plant. Note: (**E**) is attributed to [23]. Reproduced according to the terms of the Creative Commons Attribution License.

Virescence: Floral virescence refers to the abnormal greening of flower petals or other floral structures that are typically not green. For example, New Jersey aster yellows phytoplasma induces petal color change from white to green (Figure 2A). This color change is always accompanied by an increase in chlorophyll content, producing a green appearance [21].

Phyllody: Phyllody is a flower abnormality in which floral parts, such as petals, stamens, and carpels, transform into leaf-like structures. This alteration affects the flower’s ability to reproduce and alters its appearance. For instance, in potato purple top phytoplasma-infected periwinkle, the floral petals became leafy structures (Figure 2B). In some plant species, such as tomato and pepper, phytoplasma can induce big buds, a form of phyllody (Figure 2C; [8,22]). A big bud is characterized by a hollow bladder formed by the fused enlarged sepals (the outermost whorl of the flower). The flower organs in the inner three whorls, including petals, stamens, and carpels, were aborted (Figure 2C,D; [8]). The calyx/sepals occasionally opened, forming a trumpet-like structure (Figure 2D). In addition, phyllody can occur in carpels, known as carpel phylloids, which are observed in strawberries infected with phytoplasma (Figure 2E; [23]).

Cauliflower-like inflorescence (CLI): The CLI symptoms induced by phytoplasma infection typically include the formation of abnormal cauliflower-like growths on the plant’s inflorescence structures. Flower development on such inflorescences is halted (Figure 2F; [8]), leading to the failure of the fruit set, thus reducing the yield of the harvested crop.

**Figure 3:**
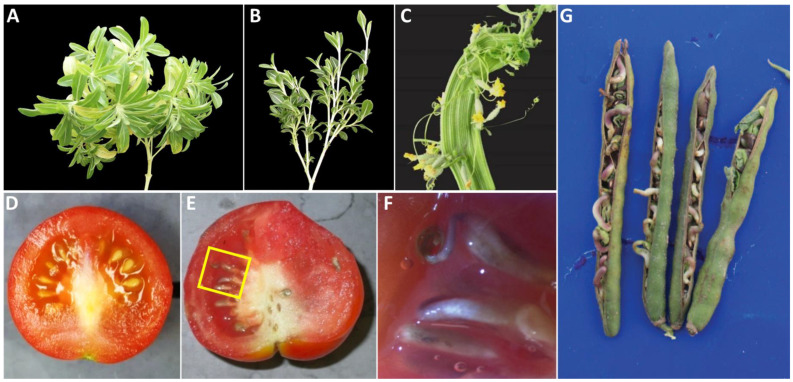
Phytoplasma infection-induced abnormalities in plants. (**A**,**B**), witches’-broom symptoms caused by different phytoplasmas in periwinkles. (**C**), phytoplasma-induced stem fasciation in cucumbers. (**D**), healthy tomato fruit and seeds for comparison. E, vivipary symptom in tomato, where seeds germinated inside the fruit. (**F**), a close-up image of a yellow box in (**E**). (**G**), vivipary symptom in mung bean, where seeds germinated inside the bean pods. Note: (**C**) is attributed to [24]; reproduced according to the terms of the Creative Commons Attribution License. (**G**) is attributed to the [25] and is used with permission from the Journal (https://ww.tandfonline.com, Taylor & Francis Ltd.).

Witches’-broom: Witches’-broom is characterized by the abnormal proliferation of shoots, resulting in a dense cluster of small branches resembling a broom. These shoots often grow from a single point on the plant, exhibiting denser growth than the rest of the plant (Figure 3A,B). Witches’-broom is frequently accompanied by little leaves, yellowing, and stunting symptoms.

### 3.4. Other Symptoms Induced by Phytoplasma Infections

Stem fasciation: Stem fasciation is abnormal growth characterized by flattening, ribboning, swelling, fusion, or elongation of the stem (Figure 3C; [24]).

Vivipary: Seeds germinate inside the fruits, which are attached to the parent plants. For example, the potato purple top (PPT) phytoplasma infection caused viviparous symptoms in an early-flowering tomato line (Figure 3D–F; [9]). Viviparous shoots were also observed in mung bean pods (Figure 3G; [25]).

### 3.5. Symptomatic Complexity Is Caused by Phytoplasma-Induced Meristem Fate Changes

Symptoms induced by phytoplasmas manifest through a range of changes in meristem fate, including early termination of the floral meristem [9], conversion of floral meristem fate from determinate to indeterminate mode [7], and even the cessation of meristematic activity [21]. These alterations often lead to visible symptoms such as witches’- broom, phyllody, virescence, and other deformities in affected plants [6,7,8,9,11]. The correlation between alterations in meristematic activity and symptom presentation is outlined in Table 1. More importantly, phytoplasma-induced symptoms sequentially develop in plants, targeting different developmental stages of the meristems and altering plant architecture and growth patterns. In other words, multiple symptoms can be observed in a single plant (for more detail, refer to Figure 2G).

### 3.6. Phytoplasma Disease and Symptom Database (iPhyDSDB)

Symptomatic images were identified from each species after finalizing the number of plant species (945 at the species level) associated with phytoplasma infections. A total of 1264 image address links were collected from 372 species. For the remaining 573 species, no symptom images were available. Such a lack of symptom images was likely caused by the following two situations: (i) phytoplasma infection indeed induced characteristic symptoms, but symptom images were not provided by the original author(s) of the report. For example, the 16SrI-B phytoplasma strain was reported to induce witches’-broom symptoms in pineapples in India, which was published as a Plant Disease Note. However, no symptom images were found [27]; (ii) phytoplasmas were detected in specific plant species/cultivars, but the infected plant(s) did not display symptoms. In Brazil, 16SrII-C phytoplasma was detected in acid lime trees, which do not exhibit symptoms [28]. In addition, certain weeds and grasses serve as reservoirs for phytoplasma transmission, but they are asymptomatic [12].

For some extensively studied phytoplasma diseases, such as Flavescence dorée (golden yellowing in French), Bois Noir (black wood in French) diseases in grapevines, and potato purple top disease in tomato plants, many symptom images are available online. For such diseases, only a few (around five) representative disease image links were collected for the database. In addition, the diseased palm hosts are often known or identified at the species level. For example, coconut palm (Cocos nucifera) was infected with 16SrIV-C phytoplasma strain in Tanzania [29]. So far, nearly 40 palm species can be infected by phytoplasmas (this study; [30]; for further detail, refer to the *i*PhyDSDB website mentioned in the next section). Different palm species exhibit similar symptoms. Therefore, only representative symptom image URLs were collected across various palm species. However, it is worth noting that some phytoplasma-infected plants, such as grapevine and citrus plants, are not consistently recognized at the species level. For example, phytoplasma infections are frequently found in various citrus hybrids, such as key lime (*Citrus × aurantifolia*) and sweet orange (*Citrus × sinensis*) [31,32]. These citrus hybrids were selected by breeders based on a few primary and core species.

### 3.7. iPhyDSDB Website

The *i*PhyDSDB database was further made accessible online through the website (https://plantpathology.ba.ars.usda.gov/iphydsdb/iphydsdb.html, accessed on 23 May 2024). This resource offers detailed information, including (i) known plant hosts at the species level with both common and scientific names, as well as plant hosts identified under the genus level (species identification has not yet been conducted); (ii) a collection of original links to symptomatic images, offering visual aids for better understanding and identification of plant diseases and making it easier for users to access the relevant information and original publications; and (iii) descriptions of typical symptoms induced by phytoplasmas, helping users recognize and diagnose issues accurately.

In addition, the website is equipped with an advanced search function that allows users to perform searches/queries using keywords, including symptom-related terms (such as virescence, phyllody, and witches’-broom) or plant hosts by common or scientific names (Figure 1). The search/query results will be displayed in a table containing the name of the phytoplasma disease, disease symptoms, and symptom image address links. This searchable function is helpful for researchers, educators, and practitioners in the field of plant pathology to locate relevant information.

## 4. Discussion

The curated symptom data and search functionality of the *i*PhyDSDB database and website contribute to the accuracy and efficiency of diagnosing phytoplasma-related diseases. The database streamlines the diagnostic process by enabling users to search and cross-reference symptoms with specific plant hosts. Additionally, the inclusion of symptom image links offers a valuable visual aid, further supporting the identification of characteristic symptoms.

A feature that allows users to submit their own images and descriptions of suspicious plant infections will be included in the *i*PhyDSDB interface so users can inquire about possible phytoplasma infections and get help from experts for confirmation. After validation, new symptom images of phytoplasma-infected plant hosts may be added to the database, thereby enriching the resource.

This study found that some plant hosts were only identified at the genus level. In the future, when reporting a new plant host, species-level identification is recommended. The plants can be determined at the species level by using plant barcoding markers such as ribulose 1,5-biphosphate carboxylase large subunit (*rbc*L), the intergenic region between chloroplast genes trnL and *trn*F (*trn*L-*trn*F), and maturase K (*mat*K) [33,34,35]. In addition, we sometimes encountered a challenge during image data collection. Some reports described the disease symptoms but did not provide the corresponding symptom images. It is recommended that the disease symptom images are submitted when reporting a new phytoplasma disease or existing diseases in new plant hosts.

When reporting new hosts of phytoplasma disease or new phytoplasma strains, the geographical distribution of diseases and hosts should be provided. If available, including the GPS coordinates of the sampling location is encouraged as well. This will enhance the understanding of spatial patterns and the spread of diseases.

Many plant species can be infected by multiple, distinct ‘*Candidatus* Phytoplasma’ species, resulting in different or sometimes indistinguishable symptoms. A sister database currently under construction will provide information on phytoplasma diversity, classification, host range, and geographic distribution.

As more images of phytoplasma disease symptoms are added to the database, the increased image data will facilitate the development of AI-based detection of phytoplasma diseases. In addition, image recognition and machine learning technology advancements can be integrated into smartphone apps and online platforms. These advancements will allow users to access information on the go and receive real-time assistance in diagnosing phytoplasma diseases. A symptom database and AI-based detection tool will help growers diagnose and manage phytoplasma diseases, minimizing yield losses and securing farmers’ livelihoods.

## 5. Conclusions

Phytoplasmas are small bacteria transmitted by insect vectors, causing significant economic losses by affecting plant growth and crop yield. Symptoms include virescence, phyllody, and witches’-broom, while some plants serve as symptomless reservoirs. Early diagnosis is crucial but challenging due to reliance on molecular techniques. Current plant disease databases lack comprehensive data on phytoplasma symptoms. We developed the Phytoplasma Disease and Symptom Database (*i*PhyDSDB), featuring nearly 1300 symptomatic image links from more than 370 plant species. This web-based resource aids in diagnosing phytoplasma diseases, allowing farmers and growers to quickly recognize symptoms and take preventive measures, thereby enhancing global efforts to manage these diseases.

## Figures and Tables

**Table 1:** Relationship between changes in meristem activity and the resulting plant growth abnormalities.

Plant Growth Disorder	Brief Description of the Disorder	Meristem Activity	Reference
Virescence	Abnormal greening of flower petals or other floral structures	Premature termination of floral meristem (FM)	[9]
Phyllody	Floral parts, such as petals, stamens, and carpels, transform into leaf-like structures	Premature termination of FM	[8,9]
Cauliflower-like inflorescence (CLI)	Abnormal, cauliflower-like growths on the plant’s inflorescence structures	Repetitive initiation of inflorescence meristems in place of floral meristems. The formation and development of FM had been suppressed at this stage. Although floral organ primordia may sometimes be visible, they hardly progress to further development.	[8,9]
Witches’-broom	Abnormal proliferation of shoots, resulting in a dense cluster of small branches resembling a broom	Repetitive initiation of lateral vegetive meristem from each leaf axil.	[8,9]
Vivipary	Precocious seed germination while still attached to the mother plant	Premature activation of the embryonic apical meristems without dormancy; affects the developmental programming of the seedling.	[9]
Stem fasciation	Abnormal growth is characterized by flattening, swelling, fusion, or elongation of the stem	Disrupted meristem cell organization and enhanced activity of apical meristem *	[26]

* Meristematic changes in phytoplasma-induced stem fasciation have not been investigated and confirmed.

## Data Availability

The present study’s data are available upon request to the corresponding author due to the privacy policy.

## References

[1] Hogenhout S.A., Oshima K., Ammar E.D., Kakizawa S., Kingdom H.N., Namba S. (2008). Phytoplasmas: Bacteria that manipulate plants and insects. Mol. Plant Pathol..

[2] Lee I.M., Davis R.E., Gundersen-Rindal D.E. (2000). Phytoplasma: Phytopathogenic mollicutes. Annu. Rev. Microbiol..

[3] Wei W., Zhao Y. (2022). Phytoplasma taxonomy: Nomenclature, classification, and identification. Biology.

[4] Weintraub P.G., Beanland L. (2006). Insect vectors of phytoplasmas. Annu. Rev. Entomol..

[5] Huang W., MacLean A.M., Sugio A., Maqbool A., Busscher M., Cho S.T., Kamoun S., Kuo C.H., Immink R.G., Hogenhout S.A. (2021). Parasitic modulation of host development by ubiquitin-independent protein degradation. Cell.

[6] Sugio A., MacLean A.M., Grieve V.M., Hogenhout S.A. (2011). Phytoplasma protein effector SAP11 enhances insect vector reproduction by manipulating plant development and defense hormone biosynthesis. Proc. Natl. Acad. Sci. USA.

[7] MacLean A.M., Sugio A., Makarova O.V., Findlay K.C., Grieve V.M., Tóth R., Nicolaisen M., Hogenhout S.A. (2011). Phytoplasma effector SAP54 induces indeterminate leaf-like flower development in Arabidopsis plants. Plant Physiol..

[8] Wei W., Davis R.E., Nuss D.L., Zhao Y. (2013). Phytoplasmal infection derails genetically preprogrammed meristem fate and alters plant architecture. Proc. Natl. Acad. Sci. USA.

[9] Wei W., Davis R.E., Bauchan G.R., Zhao Y. (2019). New symptoms identified in phytoplasma-infected plants reveal extra stages of pathogen-induced meristem fate-derailment. Mol. Plant-Microbe Interact..

[10] Wei W., Zhao Y., Davis R.E. (2019). Phytoplasma inoculum titre and inoculation timing influence symptom development in newly infected plants. Phytopathogenic Mollicutes.

[11] Wei W., Inaba J., Zhao Y., Mowery J.D., Hammond R. (2022). Phytoplasma infection blocks starch breakdown and triggers chloroplast degradation, leading to premature leaf senescence, sucrose reallocation, and spatiotemporal redistribution of phytohormones. Int. J. Mol. Sci..

[12] Zwolińska A., Krawczyk K., Borodynko-Filas N., Pospieszny H. (2019). Non-crop sources of Rapeseed Phyllody phytoplasma (‘*Candidatus* Phytoplasma asteris’: 16SrI-B and 16SrI-(B/L) L), and closely related strains. Crop Prot..

[13] Marcone C., Valiunas D., Salehi M., Mondal S., Sundararaj R. (2023). Phytoplasma diseases of trees. For. Microbiol..

[14] Wu Y., Zhang F., Yang K., Fang S., Bu D., Li H., Sun L., Hu H., Gao K., Wang W. (2019). SymMap: An integrative database of traditional Chinese medicine enhanced by symptom mapping. Nucleic Acids Res..

[15] Sharma N., Krishnan P., Kumar R., Ramoji S., Chetupalli S.R., Ghosh P.K., Ganapathy S. (2020). Coswara—A database of breathing, cough, and voice sounds for COVID-19 diagnosis. arXiv.

[16] PlantVillage Dataset. https://www.kaggle.com/datasets/emmarex/plantdisease.

[17] Namba S. (2011). Phytoplasmas: A century of pioneering research. J. Gen. Plant Pathol..

[18] Rao G.P., Rao A., Kumar M., Ranebennur H., Mitra S., Singh A.K. (2020). Identification of phytoplasma in six fruit crops in India. Eur. J. Plant Pathol..

[19] Kastal’eva T.B., Bogoutdinov D.Z., Bottner-Parker K.D., Girsova N.V., Lee I.M. (2016). Diverse phytoplasmas associated with diseases in various crops in Russia–pathogens and vectors. Agric. Biol..

[20] Rao G.P. (2021). Our understanding about phytoplasma research scenario in India. Indian Phytopathol..

[21] Inaba J., Wei W. (2024). Phytoplasma Infection-Induced Vegetative and Reproductive Abnormalities in Host Plants.

[22] Çarpar H., Sertkaya G. (2022). Investigation on phytoplasma diseases, their potential insect vectors and other hosts in pepper (*Capsicum annuum* L.) growing areas of Hatay-Turkey. Mustafa Kemal Üniversitesi Tarım Bilim. Derg..

[23] Pérez-López E. (2019). Strawberry green petal disease: Beautiful symptoms, devastating disease. Rev. Biol. Trop..

[24] Wang X., Hu Q., Wang J., Lou L., Xu X., Chen X. (2022). Comparative Biochemical and Transcriptomic Analyses Provide New Insights into Phytoplasma Infection Responses in Cucumber. Genes.

[25] Akhtar K.P., Sarwar G., Abbas G., Asghar M.J., Sarwar N., Hamed M. (2012). Mungbean phyllody disease in Pakistan: Symptomatology, transmission, varietal response and effects on yield characteristics. Int. J. Pest Manag..

[26] Iliev I., Kitin P. (2011). Origin, morphology, and anatomy of fasciation in plants cultured in vivo and in vitro. Plant Growth Regul..

[27] Mitra S., Debnath P., Bahadur A., Chandra Das S., Yadav A., Rao G.P. (2019). First report on ‘*Candidatus* Phytoplasma asteris’(16SrI-B subgroup) strain associated with pineapple shoot proliferation and witches’-broom symptoms in Tripura, India. Plant Dis..

[28] Silva F.N., Queiroz R.B., Souza A.N., Al-Sadi A.M., Siqueira D.L., Elliot S.L., Carvalho C.M. (2014). First report of a 16SrII-C phytoplasma associated with asymptomatic acid lime (*Citrus aurantifolia*) in Brazil. Plant Dis..

[29] Mpunami A., Pilet F., Fabre S., Kullaya A., Dickinson M., Dollet M. (2021). Spatial distribution of the different strains of the distinct coconut lethal yellowing-type phytoplasma species associated with the syndrome in Tanzania. Trop. Plant Pathol..

[30] Harrison N.A., Jones P. (2004). Disease caused by a phytoplasma: Lethal yellowing. Compend. Ornam. Palm Dis. Disord..

[31] Al-Yahyai R., Al-Subhi A., Al-Sabahi J., Al-Said F., Al-Wahaibi K., Al-Sadi A.M. (2014). Chemical composition of acid lime leaves infected with *Candidatus* Phytoplasma aurantifolia. Agric. Sci..

[32] Quiroga N., Gamboa C., Medina G., Contaldo N., Torres F., Bertaccini A., Zamorano A., Fiore N. (2021). Survey for ‘*Candidatus* Liberibacter’ and ‘*Candidatus* Phytoplasma’ in Citrus in Chile. Pathogens.

[33] Inaba J., Shao J., Trivellone V., Zhao Y., Dietrich C.H., Bottner-Parker K.D., Ivanauskas A., Wei W. (2023). Guilt by Association: DNA Barcoding-Based Identification of Potential Plant Hosts of Phytoplasmas from Their Insect Carriers. Phytopathology®.

[34] Staudacher K., Wallinger C., Schallhart N., Traugott M. (2011). Detecting ingested plant DNA in soil-living insect larvae. Soil Biol. Biochem..

[35] Jurado-Rivera J.A., Vogler A.P., Reid C.A., Petitpierre E., Gómez-Zurita J. (2009). DNA barcoding insect–host plant associations. Proc. R. Soc. B Biol. Sci..

